# LRP5 negatively regulates differentiation of monocytes through abrogation of Wnt signalling

**DOI:** 10.1111/jcmm.12190

**Published:** 2013-11-25

**Authors:** Maria Borrell-Pagès, July Carolina Romero, Lina Badimon

**Affiliations:** a Cardiovascular Research Center, CSIC-ICCC, Hospital de la Santa Creu i Sant Pau, IIB-Sant PauBarcelona, Spain; b CIBERobn, Fisiopatología de la Obesidad y Nutrición, Instituto de Salud Carlos IIIBarcelona, Spain; c Cardiovascular Research Chair, UABBarcelona, Spain

**Keywords:** LRP5, apoptosis, cellular differentiation, macrophages, HL60

## Abstract

Molecular changes involved in cell differentiation are only partially known. Circulating inflammatory cells need to differentiate to perform specialized functions in target tissues. Here, we hypothesized that low-density lipoprotein receptor–related protein 5 (LRP5) is involved, through its participation in the canonical Wnt/β-catenin signalling, in the differentiation process of monocytic cells. To this aim, we characterized differentiation mechanisms of HL60 cells and primary human monocytes. We show that silencing the LRP5 gene increased differentiation of HL60 cells and human monocytes, suggesting that LRP5 signalling abrogates differentiation. We demonstrate that the mechanisms behind this blockade include sequestration of β-catenin at the cellular membrane, inhibition of the Wnt signalling and increase of apoptosis. We further demonstrate the involvement of LRP5 and the Wnt/β-catenin signalling in the process because cellular differentiation can be rescued by the addition of downstream Wnt target genes to the monocytic cells.

## Introduction

Low-density lipoprotein receptor–related protein 5 (LRP5) belongs to the LDL receptor family and has been recognized as a multifunctional cell surface receptor 1. From a mechanistic standpoint, LRP5 functions as a co-receptor with the protein frizzled to bind extracellular Wnt proteins resulting in the stabilization of intracellular β-catenin. The stabilized β-catenin is free to translocate to the nucleus and bind to lymphoid enhancer binding factor/T cell factor (LEF1/TCF) to regulate the transcription of Wnt-responsive genes. We have recently demonstrated that extracellular lipid binding to LRP5 in human macrophages can trigger the Wnt signalling cascade and induce macrophage motility 2. The canonical Wnt/β-catenin signalling pathway regulates multiple biological events, including proliferation and development 3,4.

Deregulation of Wnt signalling causes alterations in inflammatory mechanisms that will ultimately lead to several types of diseases, including cancer or Alzheimer disease 5. Inflammation is a complex biological response of organs and tissues to harmful stimuli 6. It is mainly orchestrated by monocytes and macrophages that respond to extracellular signals and induce inflammatory reactions by causing the release of cytokines and other inflammation mediators 7. Although the role of Wnt signalling in the inflammatory response is poorly understood, β-catenin-dependent Wnt signalling is suggested to increase the inflammatory response to LPS in human macrophages 8. Also, the proliferative effects of the Wnt/β-catenin pathway appear to enhance wound repair/healing responses. Indeed, during the wound healing process after myocardial infarction in adult male SD rats, the activated Wnt-frizzled-Dishevelled signalling pathway seems to contribute to the myofibroblast proliferation and migration 9. Although the relevance of the Wnt signalling pathway in inflammatory processes is under constant study, the independent contribution of some of its components, specifically of LRP5, has never been addressed.

In the present paper, we have hypothesized that LRP5 negatively regulates monocytic cell differentiation. We have proven this hypothesis by investigating LRP5 function in the monocytic cell line HL60 and in primary cultures of human monocytes (HM).

## Materials and methods

A complete description of the cell lines, antibodies, reagents and techniques used in this paper can be found in the Supplemental section.

## Results

### Differentiation is increased in LRP5-silenced PMA-treated HL60 cells

**Fig 1:**
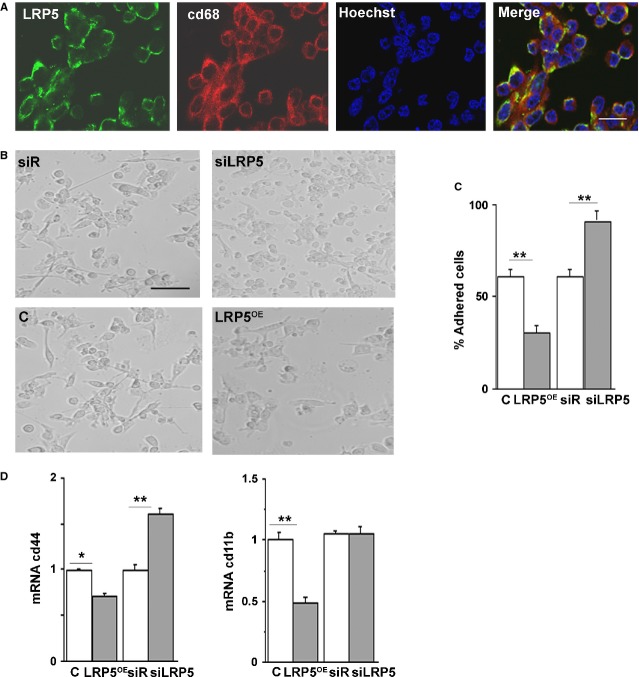
Differentiation is increased in LRP5-silenced HL60 cells. (A) Representative image of PMA-treated HL60 cells fixed, permeabilized and immunostained with mouse anti-LRP5, rabbit anti-cd68 followed by Alexa Flour anti-mouse 488 IgG, Alexa Flour anti-rabbit 633 IgG and Hoechst. Scale bar: 20 μM. (B) HL60 cells transfected to either silence (siLRP5) or overexpress LRP5 (LRP5^OE^) along with the controls (C, siR). 24 hrs after transfection, PMA (10 nM) was added to the supernatant for further 24 hrs when supernatants were collected and pictures were taken. Scale bar: 80 μM. (C) Bar graph showing the% of adhered cells in LRP5^OE^ and siRNA-LRP5 HL60 cells (D) cd44 and cd11b mRNA levels, from RNA extracts from adhered cells in B, were quantified by real time PCR and normalized to 18srRNA, **P < 0.01, *P < 0.05.

HL60 cells treated with 10 nM phorbol 12-myristate 13-acetate (PMA) differentiate to macrophages, as described previously 10,11. Immunofluorescence analysis of differentiated macrophages (measured as cd68 staining) showed expression of LRP5 (Fig. 1A). To understand the role of LRP5 in cell differentiation, we silenced its expression in undifferentiated inflammatory cells. HL60 cells were transfected with either control siRNA-Random (siR) or a siRNA against LRP5 (siRNA-LRP5), then were treated with PMA to differentiate them to macrophages (Fig. 1B, upper panels).Higher rates of cell differentiation were observed in LRP5-silenced HL60 cells (92.5 ± 3%, Fig. 1C). Undifferentiated HL60 cells were transduced with a plasmid containing LRP5 (LRP5^OE^) or the vector alone (C) and treated as above. Opposed to siRNA-LRP5 results, LRP5^OE^-HL60 cells showed lower differentiation rate, as only 22.3 ± 1% of cells were differentiated (Fig. 1B, lower panels and Fig. 1C). In these experiments, adhered and non-adhered cells were collected and viable cells were counted (Fig. S1A). Up-regulation of LRP5 reduced differentiation in a 37.5 ± 1% of cells, while silencing of LRP5 increased differentiation in a 30.3 ± 1% of cells (Fig. 1C), supporting a negative effect of LRP5 in cell differentiation. LRP5 mRNA analysis confirmed the overexpression or silencing of LRP5 in these cells (Fig. S1B).

To confirm the effects of LRP5 in cell differentiation, we analysed the expression levels of two well-established differentiation and adhesion markers, CD11b and CD44. CD11b and CD44 were down-regulated in LRP5-overexpressing cells (45 ± 3% and 38 ± 1% respectively, Fig. 1D) and CD44 was up-regulated in LRP5-silenced cells (63 ± 1%, Fig. 1D). Taken together, these results indicate that LRP5 blocks differentiation.

### LRP5 overexpression inhibits cell proliferation and induces apoptosis

**Fig 2:**
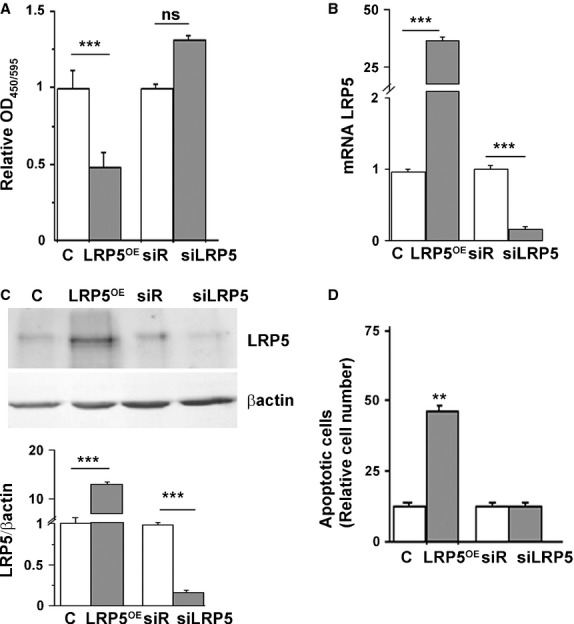
LRP5 overexpression inhibits cell proliferation and induces apoptosis in HL60 cells. (A) HL60 cells containing C, LRP5^OE^, siRNA-Random or siRNA-LRP5 were cultured in 96-well plates and 24 hrs after transfection BrdU was added for further 24 hrs. Results were read at 450–595 nm and the experiment was performed three times in duplicates. HL60 cells were transfected for 48 hrs to either silence (siLRP5) or overexpress LRP5 (LRP5^OE^) along with the controls (empty vector, C and siRNA-Random, siR) and (B) LRP5 mRNA levels from RNA extracts were quantified by real time PCR and normalized to 18srRNA. (C) Representative western blots and quantitative analysis (control cells, white boxes; LRP5-treated cells, grey boxes) of HL60-transfected cells. (D) 48 hrs post-transfection, HL60 cells were collected, washed, stained with Annexin V and PI following manufacturer's instructions and analysed by flow cytometry, **P < 0.01 all conditions respect to LRP5^OE+^. Three experiments were performed in triplicates ***P < 0.005, **P < 0.01.

We then searched for the mechanisms by which LRP5 overexpression in undifferentiated HL60 cells inhibited cell differentiation. Wnt signalling has been widely described as an inducer of cell proliferation in several cell lines, including epithelial, stromal and endothelial cells 12. HL60 proliferation, tested by measuring DNA replication by incorporation of the thymidine analogue BrdU, was inhibited by 44.5 ± 1% in LRP5-overexpressing HL60 cells (Fig. 2A, *P* < 0.005). Interestingly, LRP5 silencing did not affect cell proliferation rates. LRP5 mRNA and protein expression levels confirmed LRP5 expression levels in these cells (Fig. 2B and C).

To investigate whether the reduction in cell differentiation seen in Figure 1C and the inhibition of cell proliferation seen in Figure 2A were because of apoptotic cell death, LRP5 apoptotic features were measured by using the AnnexinV FITC/PI assay kit. Forty-eight hours after transfection, there was a threefold increase in apoptotic LRP5^OE^ cells compared with control cells (cells transfected with an empty vector, Fig. 1D, *P* < 0.01). There was no significant apoptotic cell death induced in siRNA-LRP5 cells (Fig. 1D).

### Apoptosis-related genes and proteins modulated by LRP5 expression levels

**Fig 3:**
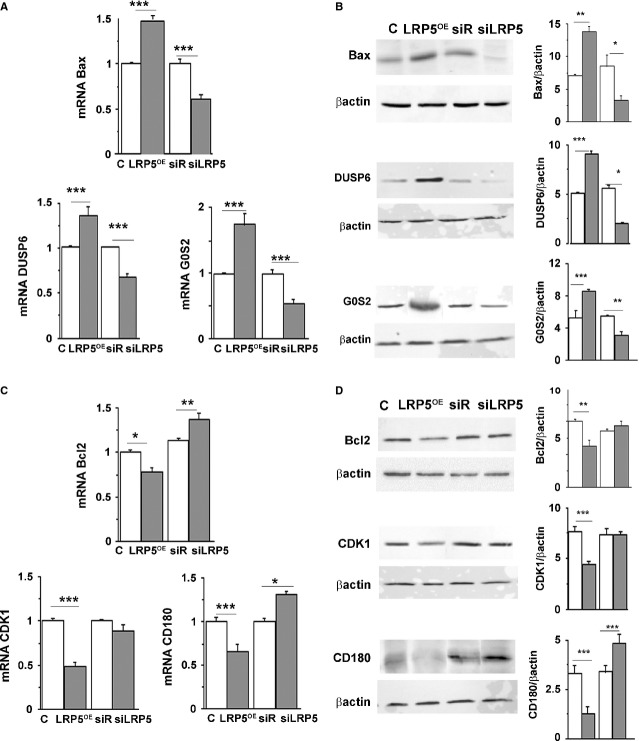
Apoptosis-related genes and proteins modulated by LRP5 expression levels. siRNA-Random, siRNA-LRP5, vector (C) and LRP5^OE^ were transfected in HL60 cells for 48 hrs and (A), mRNA levels from RNA extracts were quantified by real time PCR and normalized to 18srRNA and (B) Representative western blots and quantitative analysis of pro-apoptotic proteins Bax, DUSP6 and G0S2 were measured (control cells, white boxes; LRP5-treated cells, grey boxes). (C and D) Same as in A and B for the anti-apoptotic proteins Bcl2, CDK1 and CD180, ***P < 0.005, **P < 0.01, *P < 0.05. Experiments were performed twice in duplicates.

Apoptosis-related proteins were measured in LRP5-modified cells. We first analysed the expression levels of three pro-apoptotic genes and proteins: Bax, Dual Specific Phosphatase 6 (Dusp6) and G0/G1 switch gene 2 (G0S2). Figure 3A shows increased expression in Bax (49 ± 2%, *P* < 0.005), DUSP6 (41 ± 2%, *P* < 0.005) and G0S2 (75 ± 3%, *P* < 0.005) mRNA levels in LRP5^OE^ cells respect to C cells. LRP5 silencing not only abrogated this effect but also promoted a down-regulation of these three pro-apoptotic genes (37 ± 1%, *P* < 0.005 in Bax, 26 ± 1%, *P* < 0.005 in DUSP6 and 49 ± 3%, *P* < 0.005 in G0S2 LRP5 mRNA levels). A direct effect for LRP5 silencing and overexpression was also observed at the protein expression level for Bax, DUSP6 and G0S2 (Fig. 3B).

We also determined the levels of expression of three anti-apoptotic genes and proteins, Bcl2, Cyclin-dependent kinase 1 (CDK1) and the Toll-like receptor CD180. Cells overexpressing LRP5^OE^ had reduced mRNA levels of the anti-apoptotic proteins Bcl2 (19 ± 0.5%, *P* < 0.005), Cdk1 (52 ± 1%, *P* < 0.005) and CD180 (37.5 ± 2%, *P* < 0.005, Fig. 3C) *versus* controls. In these conditions, protein expression levels were also reduced for Bcl2, CDK1 and CD180 (Fig. 3D). LRP5-silenced cells showed an increased expression of Bcl2 and CD180 mRNA levels (24 ± 2%, *P* < 0.01 and 32 ± 1%, *P* < 0.005). Furthermore, CD180 protein levels were also increased in siRNA-LRP5-treated cells by 61 ± 3%, *P* < 0.005. Taken together, these results show increased expression levels of pro-apoptotic proteins and decreased expression levels of anti-apoptotic proteins in the presence of high levels of LRP5, supporting a key role for LRP5 in apoptotic processes.

### LRP5 silencing does not affect proliferation in adhered cancer cells

**Fig 4:**
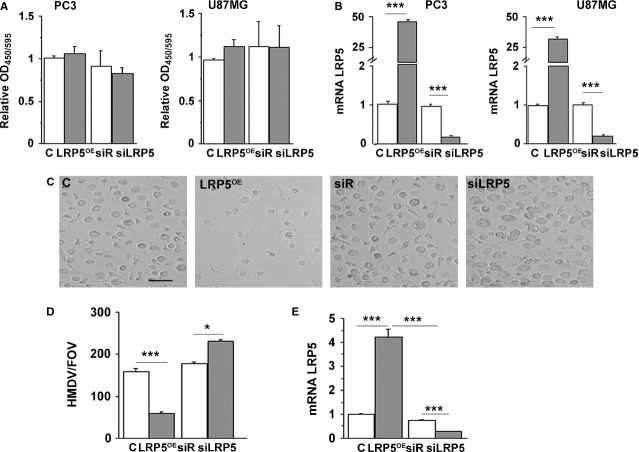
LRP5^OE^ cells inhibit HM differentiation to HMDM. (A) PC3 and U87MG cells containing C, LRP5^OE^, siRNA-Random or siRNA-LRP5 were cultured in 96-well plates and 24 hrs post-transfection BrdU was added for further 24 hrs. Results were read at 450–595 nm and the experiment was performed twice in triplicates. (B) Real time PCR quantification of LRP5 mRNA expression levels in C, LRP5^OE^, siRNA-Random and siRNA-LRP5-transfected PC3 and U87MG cells after 48 hrs. (C) HM cells transfected with vector (C), LRP5 (LRP5^OE^), siRNA-Random or siRNA-LRP5 and differentiated to HMDM when pictures were taken. Representative images of *n* = 4 experiments. Scale bar: 80 μM (D) Bar graph showing the analysis of number of HMDM/Field of Vision. (E) Real time PCR quantification of LRP5 mRNA expression levels in C, LRP5^OE^, siRNA-Random and siRNA-LRP5-transfected HM cells after 48 hrs, ***P < 0.005, *P < 0.05.

Increased adhesion and differentiation capacity in differentiation condition was observed after LRP5 silencing of HL60 cells. Therefore, we investigated whether LRP5 overexpression could also play a role in preventing proliferation of adhered cancer cells. Two common cancer cells, known for their highly proliferative profiles, were studied: prostate cancer cells (PC3) and a glioblastoma cell line (U87MG) 13–15. Neither overexpression nor silencing of LRP5 affected BrdU incorporation in either cell line (Fig. 4A). mRNA analysis was used as a control to confirm the overexpression or silencing of LRP5 (Fig. 4B).

### LRP5 silencing increases differentiation in human primary monocytes

To determine if the effect of LRP5 silencing in cellular differentiation was restrained to a monocytic cell line (HL60 cells) or was a general mechanism for undifferentiated inflammatory cells, we used HM primary cultures. Human monocytes transfected with LRP5^OE^, vector alone (C), siR or siRNA-LRP5 were spontaneously differentiated to macrophages (HMDM) in culture and analysed as before (Fig. 4C). Image analyses allowed the quantification of adhered HMDM (Fig. 4D) showing a significant reduction in adhesion of HMDM overexpressing LRP5 (166 ± 2% respect to C), while HM transfection of siRNA-LRP5 increased HM differentiation to HMDM (27 ± 1% with respect to siR). mRNA analysis was used as a control to confirm the overexpression or silencing of LRP5 (Fig. 4E). Taken together, these results further support an inhibitory role for LRP5 in monocyte to macrophage differentiation.

### Canonical Wnt pathway activation in monocytes and macrophages

**Fig 5:**
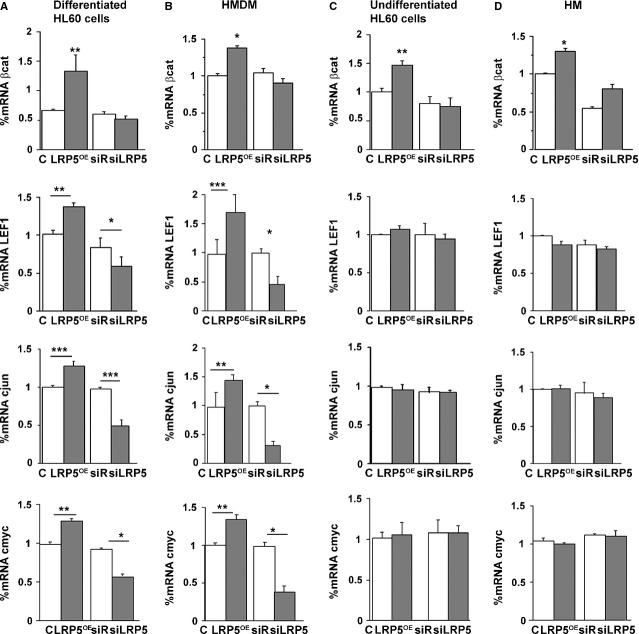
Canonical Wnt pathway activation in differentiated and undifferentiated cells. Real time PCR quantification of β-catenin, LEF1, c-jun and c-myc was performed in samples from transfected differentiated HL60 (A), differentiated HMDM (B), undifferentiated HL60 (C) and HM (D) ***P < 0.005, **P < 0.01, *P < 0.05.

Canonical Wnt signalling pathway proteins and transcription factors, including β-catenin, LEF1, c-jun and c-myc 16–18, were investigated in LRP5-modified cells. PMA-treated HL60 cells, differentiated, adherent LRP5^OE^ cells (Fig. 1C and D) showed a significant increase in β-catenin, LEF1, c-jun and c-myc mRNA expression levels (29 ± 1%, 39 ± 2%, 19 ± 2%, and 28 ± 2%, with respect to control), suggesting Wnt pathway activation (Fig. 5A). Correspondingly, LRP5-silenced cells showed a significant reduction in LEF1, c-jun and c-myc mRNA levels (33.3 ± 1%, 75 ± 1% and 48 ± 1% respectively). β-catenin gene expression levels were only slightly down-regulated. The analyses of the mRNA expression levels of β-catenin, LEF1, c-jun and c-myc in human macrophages overexpressing LRP5 revealed a 42 ± 2% increase in β-catenin, 63 ± 2% increase in LEF1, a 49 ± 1% increase in c-jun and a 32 ± 1% increase in c-myc mRNA expression levels (Fig. 5B). Again, in siRNA-LRP5 HMDM, there was a significant inhibition of LEF1, c-jun and c-myc (Fig. 5B), while β-catenin was only slightly down-regulated. Therefore, the canonical Wnt signalling pathway is activated in differentiated, PMA-treated HL60 cells and human macrophages with high LRP5 expression levels.

We then analysed the mRNA expression levels of β-catenin, LEF1, c-jun and c-myc in undifferentiated cells (HL60 and HM). β-catenin gene expression levels were increased by 49 ± 3% and 27 ± 1% in undifferentiated HL60 cells and HM respectively. However, silencing of LRP5 did not modulate β-catenin gene expression levels. Furthermore, LEF1, c-myc and c-jun were unchanged by LRP5 expression levels in undifferentiated HL60 and in HM, indicating that up-regulation or silencing of LRP5 does not affect β-catenin canonical downstream signalling (Fig. 5C and D).

### Sequestering of β-catenin to the cell membrane induced by LRP5 overexpression in undifferentiated cells

**Fig 6:**
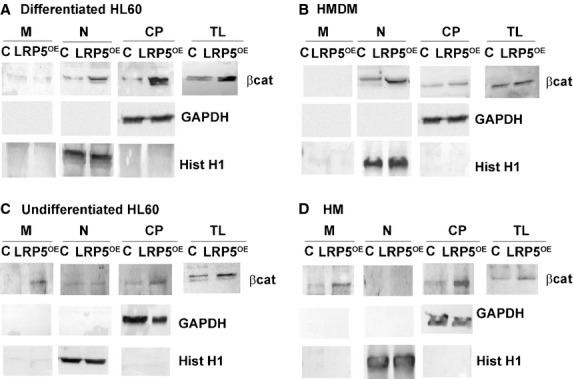
β-catenin sequestering at the plasma membrane only in undifferentiated cells. PMA-differentiated HL60 cells (A), HMDM (B), undifferentiated HL60 cells (C) or HM (D) were transfected with LRP5 (LRP5^OE^) or with the vector alone (C) and membrane (M), nuclear (N), cytoplasmic (CP) fractions and total lysates (TL) were analysed by Western blotting by using anti–β-catenin antibody. GAPDH and Histone H1 were used as quality controls for cytoplasmic/membrane and nuclear fractions respectively.

We then searched for an explanation for the results observed in the undifferentiated cells (non-differentiated, non-adhered HL60 and HM). Wnt signalling can be interrupted by sequestering β-catenin at the plasma membrane 19,20 and N-cadherin has been shown to interact with LRP5 and axin to negatively regulate Wnt signalling through β-catenin sequestration at the plasma membrane 21. By cellular subfractionation experiments, we tested whether LRP5 overexpression was antagonizing Wnt signalling in undifferentiated cells. Consistent with the current model of the canonical Wnt signalling pathway, we found that overexpression of LRP5 in differentiated cells (PMA-differentiated HL60 cells and HMDM) increased β-catenin translocation to the nucleus with respect to control cells (Fig. 6A and B). In addition, cell membrane–associated β-catenin levels did not differ in control and LRP5-overexpressing differentiated cells (Fig. 6A and B). In contrast, LRP5 overexpression in undifferentiated cells (non-differentiated, non-adhered HL60 and HM) induced little β-catenin translocation to the nucleus, while a strong β-catenin staining could be observed in the membrane fraction of LRP5-overexpressing cells (Fig. 6C and D). These results show that the defective Wnt/β-catenin signalling induced by LRP5 overexpression in undifferentiated cells results, in part, from β-catenin sequestering to the plasma membrane.

To further determine the role of LRP5 in β-catenin sequestration to the cell membrane, we performed cellular subfractionation experiments in cells without LRP5. Protein expression levels (Fig. S2) confirm gene expression levels (Fig. 5 upper panel), which shows that LRP5 depleted cells without further stimulus, do not modulate β-catenin expression levels in differentiated or undifferentiated cells. Therefore, in differentiated, adhered cells (differentiated HL60 and HMDM), β-catenin is mainly located in the cytoplasm (CP) and basal low levels of β-catenin are present in the nucleus (N). In undifferentiated cells (HL60 and HM), β-catenin is also mainly in the CP, while basal low levels are found at the plasma membrane (M). Contrarily, when LRP5 is overexpressed, Wnt signalling pathway is triggered.

### c-myc or c-jun overexpression rescue differentiation in inflammatory cells

To further determine the involvement of LRP5 in cellular differentiation, we performed rescue experiments. We have shown that LRP5 overexpression in undifferentiated cells inhibits cellular differentiation (Fig. 1), increases cellular apoptosis (Fig. 2) and inhibits canonical Wnt pathway activation (Fig. 5) by the sequestering of β-catenin to the plasma membrane (Fig. 6). We now sought to determine if an increased expression of Wnt target genes that are downstream to β-catenin's translocation to the nucleus, and may induce a constitutive activation of the canonical Wnt signalling pathway, could rescue cellular differentiation.

**Fig 7:**
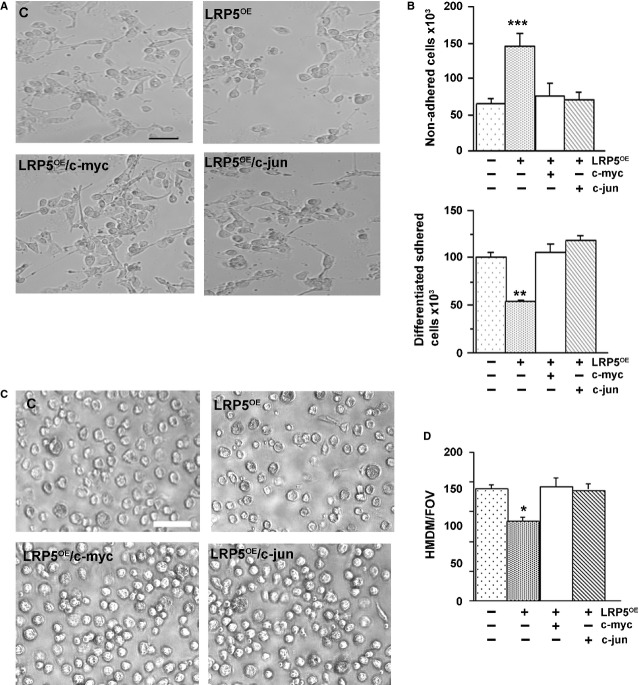
Overexpression of Wnt target genes rescues cellular differentiation. (A) 24 hrs after transfection, HL60 cells containing C, LRP5^OE^, LRP5^OE^/c-myc or LRP5^OE^/c-jun had 10 nM PMA added to the supernatant for further 24 hrs when supernatants were collected and pictures were taken. Scale bar: 80 μM. Representative images of *n* = 3 experiments (B) HL60 non-adhered and adhered cells were counted. Experiments were performed three times in triplicates, ***P < 0.005, **P < 0.01 (C) HM cells transfected with C, LRP5^OE^, LRP5^OE^/c-myc or LRP5^OE^/c-jun and differentiated to HMDM when pictures were taken. Representative images of *n* = 4 experiments.Scale bar: 40 μM (D) Bar graph showing the analysis of number of HMDM/Field of Vision ***P < 0.005, **P < 0.01, *P < 0.05.

Undifferentiated HL60 cells were transduced with control, LRP5^OE^, LRP5^OE^/c-myc or LRP5^OE^/c-jun plasmids and differentiated with PMA to macrophages (Fig. 7A). Morphometric analyses show an increase in HL60 differentiation in LRP5^OE^/c-myc and LRP5^OE^/c-jun cotransfected cells. Differentiated (adhered, Fig. 7B upper panel) and non-differentiated (non-adhered, Fig. 7B lower panel) cells were collected and counted showing an increased adhesion in LRP5^OE^/c-myc HL60 and LRP5^OE^/c-jun HL60 cells (49 ± 1% and 61 ± 1% respectively). mRNA analysis was used as a control to confirm the overexpression of c-myc and c-jun in these cells (data not shown).

Similar results were observed when HM were transfected with LRP5^OE^, LRP5^OE^/c-myc or LRP5^OE^/c-jun and differentiated to HMDM (Fig. 7C). Overexpression of LRP5^OE^/c-myc or LRP5^OE^/c-jun rescued HMDM differentiation by 28.5 ± 1% and 35.7 ± 0.5%, respectively, further demonstrating the negative regulation of LRP5 in cellular differentiation (Fig. 7D).

## Discussion

In the present work, we have addressed the effect of LRP5 modulation and downstream activation of the Wnt signalling pathway in cell proliferation, adhesion and differentiation of monocytic cells. First, we have investigated the effect of LRP5 on cell differentiation in a monocytic cell model that requires PMA for differentiation to macrophages. The LRP5 levels of pro-myelocytic leukaemia cells (HL60) were genetically modified by silencing (siRNA-LRP5) or overexpression (LRP5^OE^) experiments. siRNA-LRP5 undifferentiated HL60 cells, induced by PMA, showed increased cellular differentiation, suggesting that LRP5 could be a negative differentiation regulator of cellular differentiation. Indeed, LRP5^OE^ HL60 cells, induced by PMA, showed less differentiation than control cells and the differentiation markers CD11b and CD44 were significantly reduced. The expression of the CD11b adhesion molecule is a maturation marker for HL60 cells in response to differentiation, as more than 90% of cells became CD11b-positive following PMA treatment 11. On the other hand, the adhesion molecule CD44 is a cell surface transmembrane glycoprotein and its major physiological role is to maintain organ and tissue structure *via* cell–cell and cell–matrix adhesion (for a review, see Slevin *et al*. 22). These results strongly suggest a negative role for LRP5 in HL60 PMA-induced differentiation that may be orchestrated by LRP5′s direct or indirect interference with cellular adhesion molecules (Fig. S3).

Low-density lipoprotein receptor–related protein 5 is frequently characterized as a Wnt/β-catenin signalling activator that increases cell proliferation 23. Surprisingly, LRP5^OE^ HL60 undifferentiated cells showed decreased proliferation, as determined by decreased BrdU incorporation. The activity of the signalling components of the Wnt pathway can either foster or restrain the processes of apoptosis, according to specific cellular environment stimuli 24,25. Wnt signalling regulates the early and late stages of apoptosis in both development and adult cell injury in neurons, endothelial cells, vascular smooth muscle cells and cardiomyocytes 26. Thus, another explanation for the observed inhibition of LRP5^OE^ HL60 cell proliferation could be the activation of the apoptosis machinery. Indeed, increased apoptosis was observed in undifferentiated LRP5^OE^ HL60 cells, while siRNA-LRP5 cells did not vary their apoptotic ratio. This result is supported by studies with LRP5^−/−^ mice, where osteoblast apoptosis was found to be reduced by lithium therapy in cultures of calvaria cells derived from LRP5^−/−^ mice *ex vivo* 27, and the persistent embryonic eye vascularization of LRP5^−/−^ mice as a result of a failure of macrophage-induced endothelial cell apoptosis 28.

Our results show that an increase in LRP5 cellular expression is accompanied of increased expression levels in other pro-apoptotic proteins, including Bax, DUSP6 and G0S2. Bax is a pro-apoptotic member of the Bcl-2 family that interferes with mitochondrial function by forming pores at the outer mitochondria membrane 29. Dusp6 has been shown to participate in the pro-apoptotic pathway as its down-regulation mediates up-regulation of ERK (Extracellular signal-Regulated Kinase) and survival of endothelial cells by means of anti-apoptosis 30. Moreover, analyses by flow cytometry and immunocytochemistry revealed that the exogenous expression of Dusp6 induced apoptosis in cultured pancreatic cancer cells 31. G0S2 encodes a mitochondrial protein that specifically interacts with Bcl-2 and promotes apoptosis by preventing the formation of protective Bcl-2/Bax heterodimers 32. Interestingly, the expression levels of three broad anti-apoptotic proteins, Bcl2, Cdk1 and CD180, were significantly reduced in cells overexpressing LRP5. Bcl2 is a well-known pro-survival protein that has an essential function in a wide variety of processes (for a review, see Kelly and Strasser 33). Cdk1 interacts with cyclin B1 to form the ‘mitosis-promoting factor’ whose activity influences various pro-survival signalling pathways before entering mitosis 34,35. Also, inhibition of cyclin B1/Cdk1 activity has been shown to increase apoptosis in human tumour cells 36,37. The CD180 molecule is the transmitter of the activation signal that leads to massive proliferation as well as resistance against apoptosis 38,39. These results further support a role for high LRP5 expression levels in promoting apoptosis in undifferentiated HL60 cells.

We then analysed the highly proliferative cancer cells PC3 and U87MG to test if LRP5 overexpression could inhibit their proliferation. LRP5^OE^ PC3 or LRP5^OE^ U87MG cells did not show reduced proliferation rates, restricting the observed LRP5^OE^ role in proliferation inhibition to undifferentiated, non-adhered cells.

To confirm the inhibitory role of LRP5 overexpression in differentiation processes, we used HM primary cultures. LRP5 overexpression in HM reduced differentiation to macrophages. Wnt signalling target genes (LEF1, c-jun and c-myc) did not modulate their expression levels in LRP5^OE^ or siRNA-LRP5 HM. However, β-catenin gene expression levels were increased in the presence of LRP5^OE^, indicating a disruption in Wnt signalling downstream of β-catenin. Contrarily, adherent, differentiated LRP5^OE^ macrophages showed a significant increase in the mRNA expression levels of these Wnt genes. PMA-treated undifferentiated HL60 cells that overexpress LRP5 show that, although most of the cells do not adhere, the ones that do attach (and will eventually differentiate to macrophages) show an incipient activation of the Wnt signalling pathway. Interestingly, LRP5 overexpression was shown to inhibit osteogenic differentiation in mesenchymal stem cells 40 and repression of canonical Wnt signalling has been described in poorly differentiated hepatocellular carcinoma cells 41. Taken together, these results strongly suggest an activation of the Wnt signalling pathway only in LRP5^OE^ differentiated cells (Fig. S3).

To understand the mechanisms underlying the inhibition of cellular differentiation by LRP5^OE^, we performed subfractionation experiments that showed that LRP5^OE^ maintains undifferentiated cells in an undifferentiated state by sequestering β-catenin at the plasma membrane. It has been reported in osteoblasts that N-cadherin association with β-catenin at the membrane forms a complex with axin and LRP5 cytoplasmic tail domain causing increased β-catenin degradation, blocking canonical Wnt signalling and resulting in defective osteoblast function 21. We have demonstrated a similar process in undifferentiated monocytic cells. When cells are differentiated, LRP5 overexpression induces β-catenin translocation to the nucleus and increased expression levels of Wnt signalling genes (Fig. S3).

To further demonstrate that LRP5 is essential for cellular differentiation, we performed experiments to rescue cellular differentiation. To this end, we transfected Wnt target genes in undifferentiated cells that rescued differentiation indicating that the inhibition of the Wnt pathway and cellular differentiation in undifferentiated inflammatory cells signals *via* LRP5 (Fig. S3).

One major question raised in this study is the significance of an increased LRP5 expression in the absence of known LRP5 ligands. Several reports have shown that, in the absence of LRP5 ligands, overexpression of LRP5 lacking either the extracellular or both the transmembrane and extracellular domains leads to the activation of the Wnt signalling pathway by cytoplasmic dimerization of the receptor 3,42,43. This may explain our results in undifferentiated cells that show modulation of different biological processes in the presence of excess full-length LRP5 cDNA construct in the absence of known LRP5 ligands.

In summary, our results indicate a fine regulatory role for LRP5 in undifferentiated cells' fate. Usually, LRP5 binds its ligands (glycoproteins, extracellular lipids) and moderately increases its expression levels to activate the Wnt pathway machinery 2,44,45. In this report, we show that when undifferentiated, non-adherent cells overexpress LRP5 there is inhibition of cell proliferation and activation of the apoptosis machinery. Furthermore, there is a down-regulation of the differentiation processes by the sequestration of β-catenin to the cell membranes. The inhibition of the Wnt signalling pathway can be rescued by the addition of exogenous Wnt target genes. This is the first time that the mechanism by which LRP5 shuts off the Wnt signalling pathway to maintain cells in an undifferentiated state is described.

## Supporting information

Figure S1 HL60 cells transfected to either silence (siLRP5) or overexpress LRP5 (LRP5^OE^) along with the controls (C, siR).

Figure S2 LRP5 silencing in differentiated and undifferentiated cells.

Figure S3 Schematic showing different LRP5-β-catenin regulation in differentiated and undifferentiated cells.
