# Supplementary figures and images for: LRP5 negatively regulates differentiation of monocytes through abrogation of Wnt signalling
Source: J Cell Mol Med. 2013 Nov 25;18(2):314–25. doi: 10.1111/jcmm.12190 (PMC3930418; doi:10.1111/jcmm.12190)

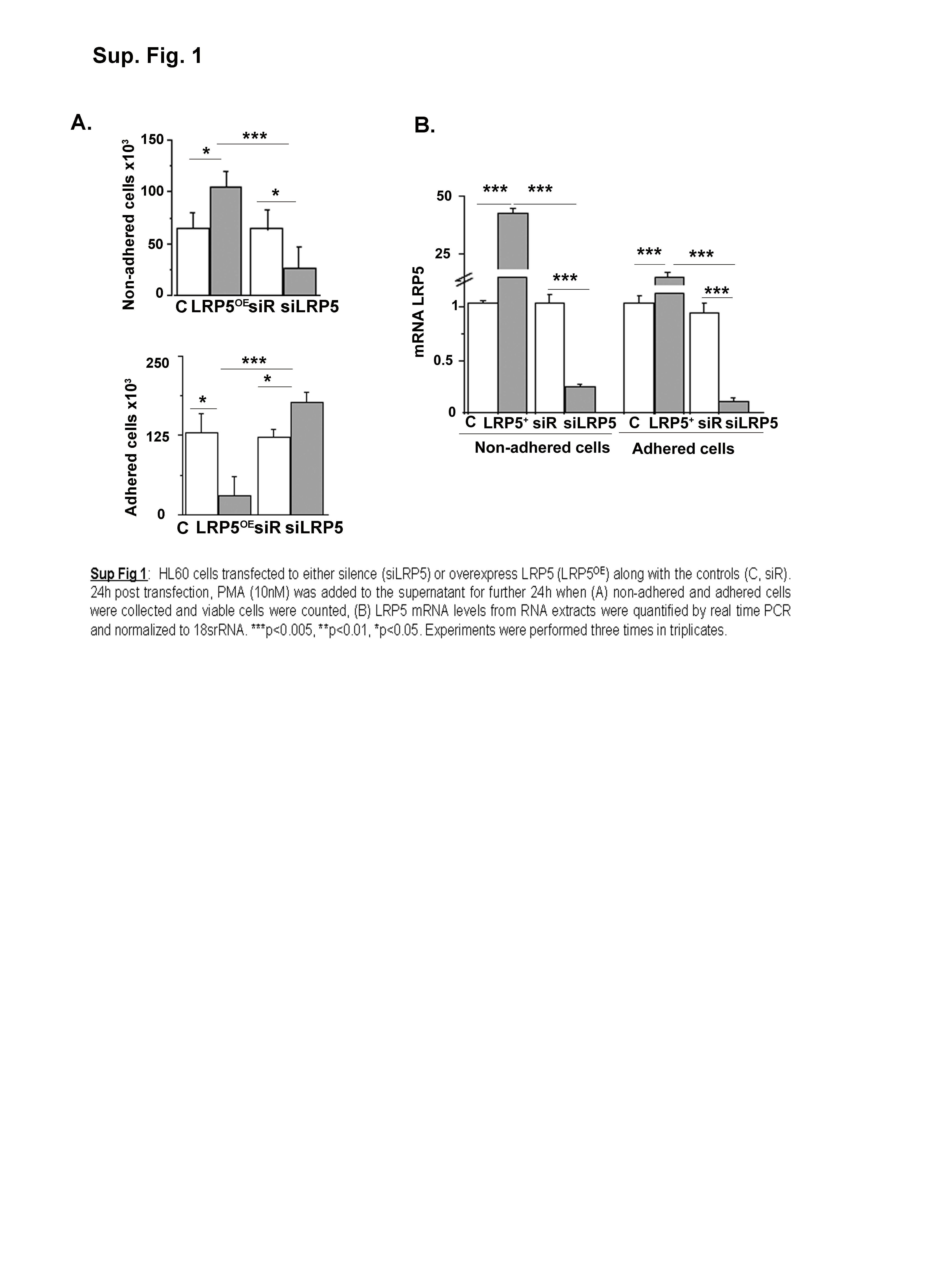

Supplement: Figure S1 — HL60 cells transfected to either silence (siLRP5) or overexpress LRP5 (LRP5OE) along with the controls (C, siR). [file jcmm0018-0314-sd1.tif]

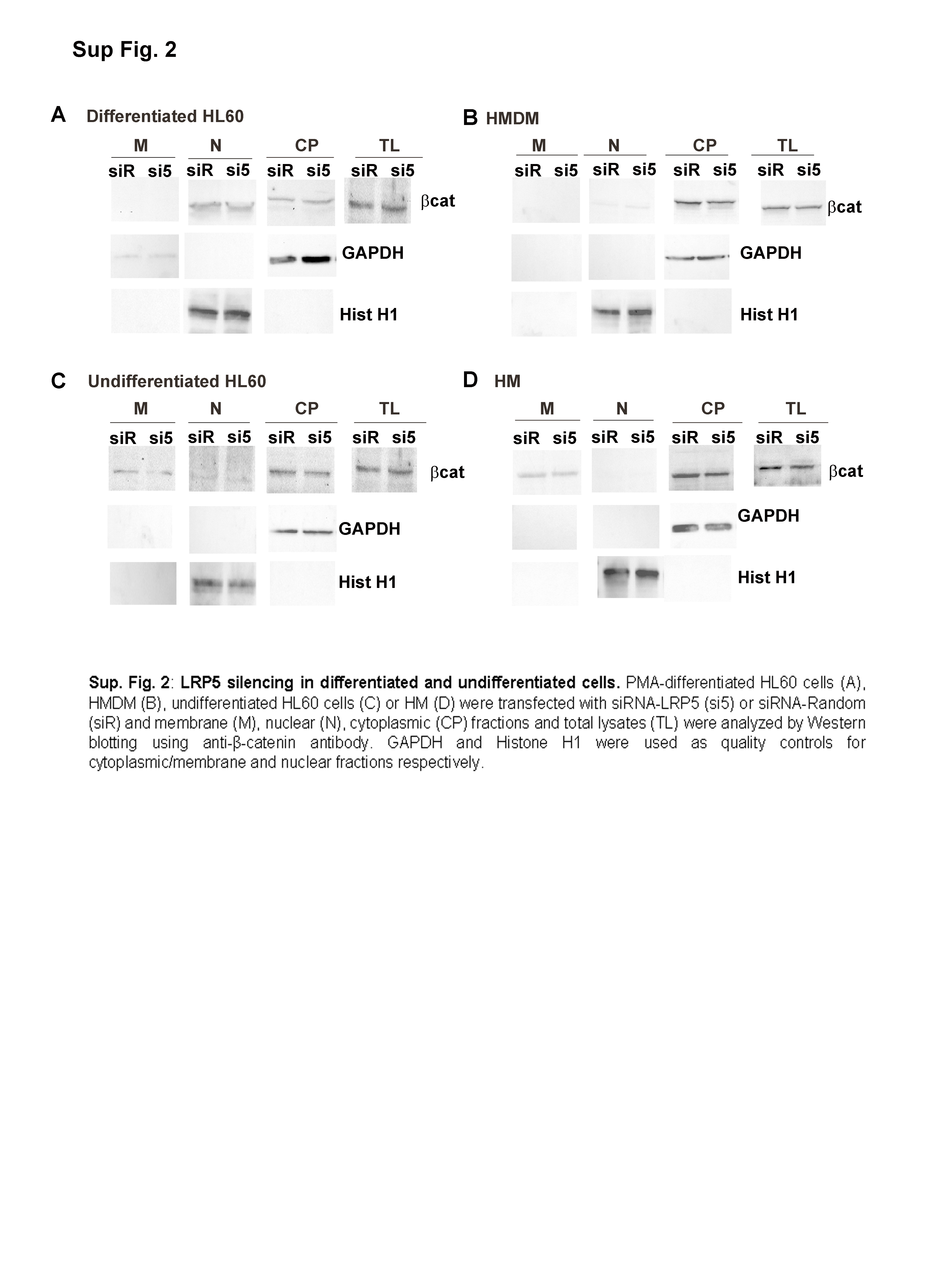

Supplement: Figure S2 — LRP5 silencing in differentiated and undifferentiated cells. [file jcmm0018-0314-sd2.tif]

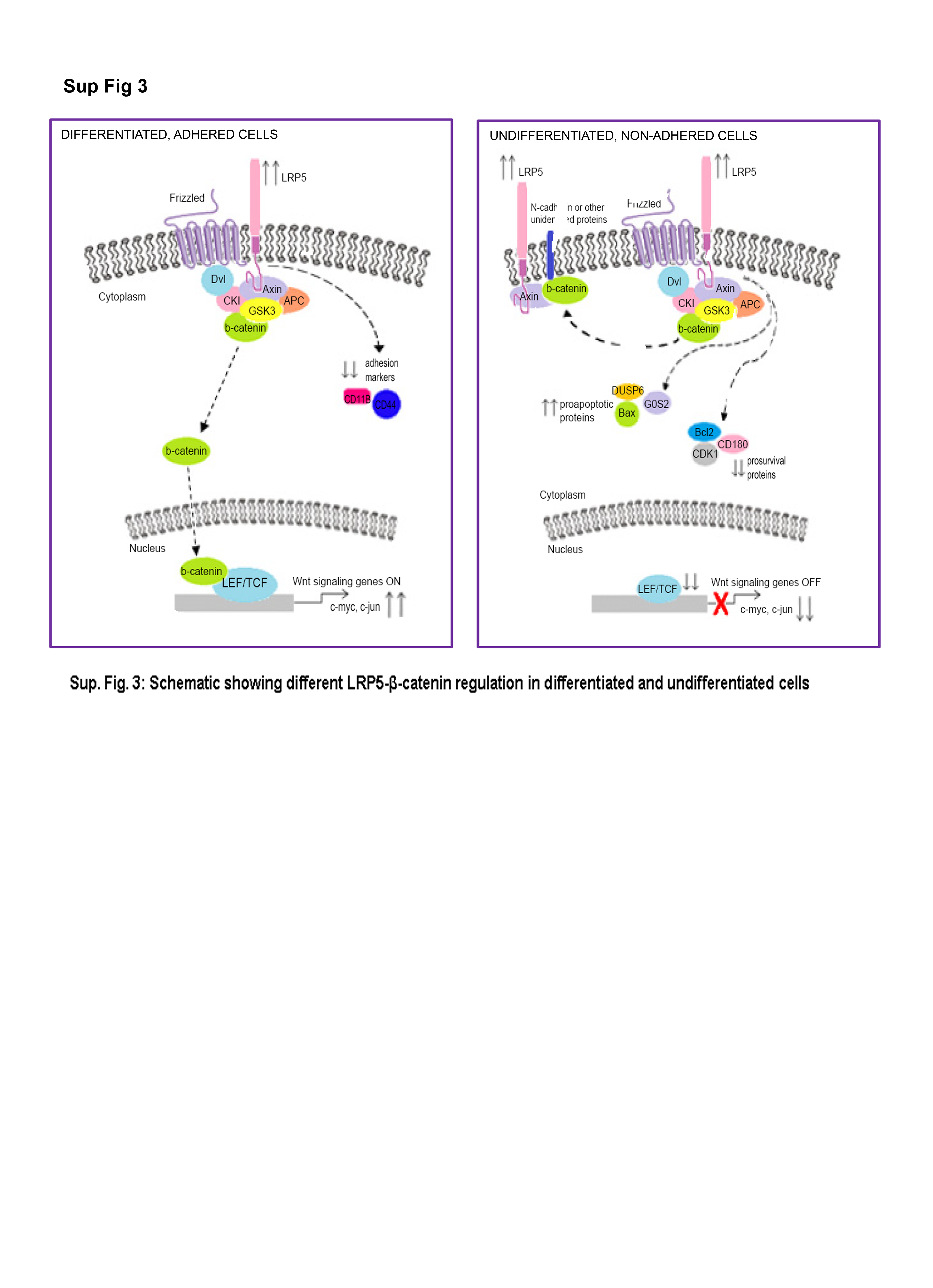

Supplement: Figure S3 — Schematic showing different LRP5-β-catenin regulation in differentiated and undifferentiated cells. [file jcmm0018-0314-sd3.tif]
