# Supplementary material for: LRP5 negatively regulates differentiation of monocytes through abrogation of Wnt signalling
Source: J Cell Mol Med. 2013 Nov 25;18(2):314–25. doi: 10.1111/jcmm.12190 (PMC3930418; doi:10.1111/jcmm.12190)
Supplement: Data S1 — Materials and methods. [file jcmm0018-0314-sd4.doc]

**Supplemental materials and methods:**

***Cell culture***

Human promyelocytic leukaemia cells (HL60) were grown in RPMI 1640 medium with Glutamax, supplemented with 10% heat-inactivated foetal bovine serum, 100U/mL penicillin and 100U/mL streptomycin during 3 days before treatment. When the culture reached a density of 7-8x10^5^ cells/mL, cells were washed, subcultured in 6 well plates (5x10^5^cells/well) and treated with PMA (10nM). 48 hours after treatment, cells achieved a macrophage phenotype, although the phagocytic function remained untested.

Human peripheral blood mononuclear cells (PBMCs) were obtained by standard protocols from buffy coats (15-20ml) from healthy donors. Cells were applied on 15 ml of Ficoll-Hypaque and centrifuged at 300g for 1 hour at 22°C, with no brake. Mononuclear cells were obtained from the central white band of the gradient, washed in phosphate buffer saline (PBS), and suspended in RPMI medium (Gibco) supplemented with 10% human serum AB (Immunogenetics) and antibiotics. Cells were seed in 6 well plates (4x10^6^cells/well) and allowed to differentiate into macrophages by 7 days in culture, replacing medium each two days..

PC3 and U87MG were grown in DMEM/F-12 medium with 10%FBS, 2mM glutamine, 100U/mL P/S.

***HL60, HM and HMDM silencing and overexpression.***

4x10^6^ HL60-cells/mL in 100µL of Cell Line Nucleofector® Solution V and 300nM of siR, siLRP5 or 0.4ug of pcDNA3 or pcDNA3-LRP5 were transfected with T-019 program.siR and pcDNA3 alone were used as controls and did not exert any effect on LRP5 expression. HM cells were nucleofected as described for HL60 cells with 0.25ug of cDNA, 1x10^7^cells/mL and X-001 transfection program.HMDM cells, PMA-treated HL60 cells, PC3 and U87MG were transfected using Metafectene® Easy^+^ (Biontex) following the manufacturer’s instructions.For silencing experiments, 100nM of each siRNA was transfected using HiPerFect Transfection Reagent (Quiagen).48 hours after transfection efficiency was systematically analyzed by LRP5 RT-Real Time PCR and normalized to r18S.

***RNA isolation and Real time PCR***

RNA was isolated using Total RNA extraction kit (Qiagen).Concentration was determined with a NanoDrop ND-1000 spectrophotometer (NanoDrop Technologies) and purity was checked by the A260/A280 ratio.cDNA was synthesized from 0.5 μg RNA with cDNA Reverse transcription kit (Qiagen). The resulting cDNA samples were amplified by PCR using a DNA thermal cycler (MJ Research) and the following specific probes from Applied Biotechnologies:LRP5 (Hs00182031-m1),18S rRNA (4319413E),CDK1 (Hs00938777-m1),G0S2 (Hs00274783-s1),CD180 (Hs01069872),DUSP6 (Hs00169257-m1),CD11b (Hs00355885-m1),CD44 (Hs01075861-m1),BAX (Hs00180269-m1),BCL2 (Hs00608023-m1),c-myc (Hs00153408_m1),c-jun (Hs99999141_s1),LEF1 (Hs01547250_m1).

***Western Blot***

Transfected HL60 cells were lysed in RIPA buffer containing protease inhibitor complex (Roche, Germany).Electrophoresis of SDS-polyacrylamide gels,transfer to nitrocellulose filters,incubation with primary antibodies (LRP5, Bcl2 and β-actin from Abcam, Cdc2p34, Bax, G0S2 and Cd180 from Santa Cruz, Dusp6 from R+D Systems) and with anti-mouse, anti-rabbit or anti-goat secondary antibodies (Dako) was performed.Band densities were determined with the ChemiDoc XRS system (Bio-Rad).Normalization was performed against β-actin.

***Bromideoxyuridine (BrdU) labeling and detection***

24 hours post-transfection BrdU (BrdU Cell Proliferation Assay, Calbiochem) was added (20uL/well of a 1:2000 dilution) for further 24 hours.Cells were fixed, incubated with anti-BrdU antibody,washed and incubated with peroxidise Goat anti-mouse IgG HRP conjugate.Substrate and stop solution were added and intensity was quantified using a spectrophotometric plate reader at dual wavelength of 450–595 nm.

***Annexin V-FITC determination***

48 hours post-transfection HL60 cells were collected, washed, stained with Annexin V fluorescein isothiocyanate (AV-FITC) and propidium iodide (PI) following manufacturer's instructions (BD Pharmingen) and analyzed by flow cytometry (FACSCalibur).

***Immunofluorescence***

PMA-treated HL60 were fixed with 4% paraformaldehyde and primary LRP5 (Biovision) and cd68 (Abcam) antibodies were added followed by Alexa Flour anti-mouse 488 IgG, Alexa Flour anti-rabbit 633 IgG and Hoechst (33342).Fluorescent images were acquired in a scan format of 1024x1024 pixels in a spatial data set (xyz) and were processed with the Leica Standard Software TCS-AOBS.Controls without primary antibodies showed no fluorescence labeling.

***Adhesion assay***

HL60 cells transfected with siRNA-Random, siRNA-LRP5, pcDNA3, pcDNA3-LRP5, pcDNA3-cmyc or c-jun were seeded in triplicates and treated with 10nM PMA for further 24h when supernatant and cell lysate were collected.A set of cells was counted on Neubahuer chambers while another set was used for RNA extraction.

***Differentiation assay in HM.***

One day after isolation, monocytes were nucleofected as described for HL60 cells with 1x10^7^cells/mL and X-001 transfection program. Cells were differentiated to HMDM for 7 days when pictures were taken and cells were counted.Transfection efficiency was analyzed 48 hours after transfection by LRP5 and r18S RT-PCR.

***Subfractionation experiments***

For nuclear extracts, cells were PBS washed,scraped with CSK buffer (50nM NaCl, 10mM Pipes pH6.8, 3mM MgCl_2_, 0.5% Triton X-100, 300mM sucrose and protease inhibitors), incubated 20 minutes shaking at 4ºC and centrifuged.Supernatants (cytoplasmic fractions) were stored at -20ºC.Pellets (nuclear fractions) was suspended in 50uL of Sol/Insol Buffer (15mM pH7.5, 5mM EDTA, 2.5mM EGTA and 1% SDS) and heated for 10 minutes at 100ºC.Membrane fraction isolation was performed as described^42^.Subcellular fractions were analyzed by western blot for β-catenin, GAPDH,Histone H1.

***Statistical analysis***

Results are expressed as mean ± S.E.M. A Stat View statistical package was used for all the analysis. When possible, comparisons among groups were performed by parametric (one factor ANOVA) analysis. Statistical significance was considered when p<0.05.

**Supplemental Figure legends:**

**Sup. Fig.** **1**: HL60 cells transfected to either silence (siLRP5) or overexpress LRP5 (LRP5^OE^) along with the controls (C, siR). 24h post transfection, PMA (10nM) was added to the supernatant for further 24h when (A) non-adhered and adhered cells were collected and viable cells were counted, (B) LRP5 mRNA levels from RNA extracts were quantified by real time PCR and normalized to 18srRNA. ***p<0.005, **p<0.01, *p<0.05. Experiments were performed three times in triplicates.

**Sup. Fig. 2**: **LRP5 silencing in differentiated and undifferentiated cells.** PMA-differentiated HL60 cells (A), HMDM (B), undifferentiated HL60 cells (C) or HM (D) were transfected with siRNA-LRP5 (si5) or siRNA-Random (siR) and membrane (M), nuclear (N), cytoplasmic (CP) fractions and total lysates (TL) were analyzed by Western blotting using anti-β-catenin antibody. GAPDH and Histone H1 were used as quality controls for cytoplasmic/membrane and nuclear fractions respectively.

**Sup. Fig. 3**: Schematic showing different LRP5-β-catenin regulation in differentiated and undifferentiated cells.
